# Network Pharmacology to Uncover the Biological Basis of Spleen Qi Deficiency Syndrome and Herbal Treatment

**DOI:** 10.1155/2020/2974268

**Published:** 2020-08-27

**Authors:** Xin Wang, Min Wu, Xinxing Lai, Jiahui Zheng, Minghua Hu, Yan Li, Shao Li

**Affiliations:** ^1^MOE Key Laboratory of Bioinformatics, Bioinformatics Division and Center for TCM-X, BNRIST / Department of Automation, Tsinghua University, Beijing 100084, China; ^2^Infinitus (China) Company Ltd., LKK Health Products Group, 510623, China; ^3^State Key Laboratory of Bioactive Substances and Functions of Nature Medicines, Institute of Materia Medica, Chinese Academy of Medical Sciences & Peking Union Medical College, 100050 Beijing, China

## Abstract

Spleen qi deficiency (SQD) syndrome is one of the basic traditional Chinese medicine (TCM) syndromes related to various diseases including chronic inflammation and hypertension and guides the use of many herbal formulae. However, the biological basis of SQD syndrome has not been clearly elucidated due to the lack of appropriate methodologies. Here, we propose a network pharmacology strategy integrating computational, clinical, and experimental investigation to study the biological basis of SQD syndrome. From computational aspects, we used a powerful disease gene prediction algorithm to predict the SQD syndrome biomolecular network which is significantly enriched in biological functions including immune regulation, oxidative stress, and lipid metabolism. From clinical aspects, SQD syndrome is involved in both the local and holistic disorders, that is, the digestive diseases and the whole body's dysfunctions. We, respectively, investigate SQD syndrome-related digestive diseases including chronic gastritis and irritable bowel syndrome and the whole body's dysfunctions such as chronic fatigue syndrome and hypertension. We found innate immune and oxidative stress modules of SQD syndrome biomolecular network dysfunction in chronic gastritis patients and irritable bowel syndrome patients. Lymphocyte modules were downregulated in chronic fatigue syndrome patients and hypertension patients. From experimental aspects, network pharmacology analysis suggested that targets of *Radix Astragali* and other four herbs commonly used for SQD syndrome are significantly enriched in the SQD syndrome biomolecular network. Experiments further validated that *Radix Astragali* ingredients promoted immune modules such as macrophage proliferation and lymphocyte proliferation. These findings indicate that the biological basis of SQD syndrome is closely related to insufficient immune response including decreased macrophage activity and reduced lymphocyte proliferation. This study not only demonstrates the potential biological basis of SQD syndrome but also provides a novel strategy for exploring relevant molecular mechanisms of disease-syndrome-herb from the network pharmacology perspective.

## 1. Introduction

Understanding the biological basis of syndromes (“ZHENG” in Mandarin Chinese) is an essential component of traditional Chinese medicine (TCM) modernization. Spleen qi deficiency (SQD) syndrome is one of the most common TCM syndromes and is characterized by fatigue, abdominal distension, boredom, and other phenotypes [1, 2]. However, the current research of SQD syndrome is somewhat limited [3] and may not be suitable for elucidating the biological basis of SQD syndrome at a systematic level. Some studies have explored the biological basis of some typical syndromes in TCM such as Cold syndrome and Hot syndrome. Based on network pharmacology analysis, Cold syndrome and Hot syndrome are closely associated with the metabolism-immune imbalance [4]. The biological networks underlying Cold syndrome and Hot syndrome have been applied to clinical investigation by integrating clinical transcriptional profiles. The network modules underlying Cold syndrome indicate that energy metabolism decreased in Cold syndrome patients [5]. In addition, the Hot syndrome biomolecular network suggests that inflammatory response increased in chronic gastritis with Hot syndrome [6]. These findings provide an important foundation at the molecular and systematic level for studying other typical syndromes such as SQD syndrome.

SQD syndrome-related research mainly contains clinical observations and experiments. In clinical terms, according to the holistic perspective of TCM, SQD syndrome not only refers to local digestive diseases such as chronic gastritis and irritable bowel syndrome but also contains the whole body's dysfunctions such as chronic fatigue syndrome and hypertension [7–9]. In experimental terms, animal models have been constructed by reserpine injection or irregular food administration [10]. Understanding the biological basis of SQD syndrome may guide the evidence-based clinical use of herbal formulae. Herbal formulae such as Si-Jun-Zi decoctions in China's National Basic Medical Insurance Drug Catalogue are widely used clinically to treat chronic gastritis and other SQD syndrome-related diseases [11, 12]. Most of the studies on these herbal formulae have focused on immunity, antioxidation, and metabolism [13–15]. Revealing the biological basis of SQD syndrome from a systematic perspective will be of great help to precision medicine for TCM syndrome-related diseases.

Given the advent of the artificial intelligence and big data era, the rapid development of information science and omics technology has provided a solid foundation for moving beyond the limitations of current medical research methods and establishing new network-based approaches [4, 16]. In recent years, the “network target, multicomponent therapeutics” approach was proposed for investigating complex diseases and herbal formulae [17]. A network target is a core principle in the network pharmacology. Different from the “one target, one drug” paradigm, the network target refers to a novel concept that treats the biological network underlying diseases and TCM syndromes as a therapeutic target in order to decipher systematic mechanisms of action for multitarget drugs and herbal formulae [18]. More and more evidence shows that the network target approach is suitable for elucidating the biological basis of the TCM syndrome and herbal treatment. For example, the network target approach is used to detect antirheumatic mechanisms of the TCM formula Qing-Luo-Yin for treating Hot syndrome-related rheumatoid arthritis [19] and unveil the molecular mechanisms of Ge-Gen-Qin-Lian decoction, which is an ancient and effective herbal formula for “dampness heat” syndrome type II diabetes [20].

**Figure 1:**
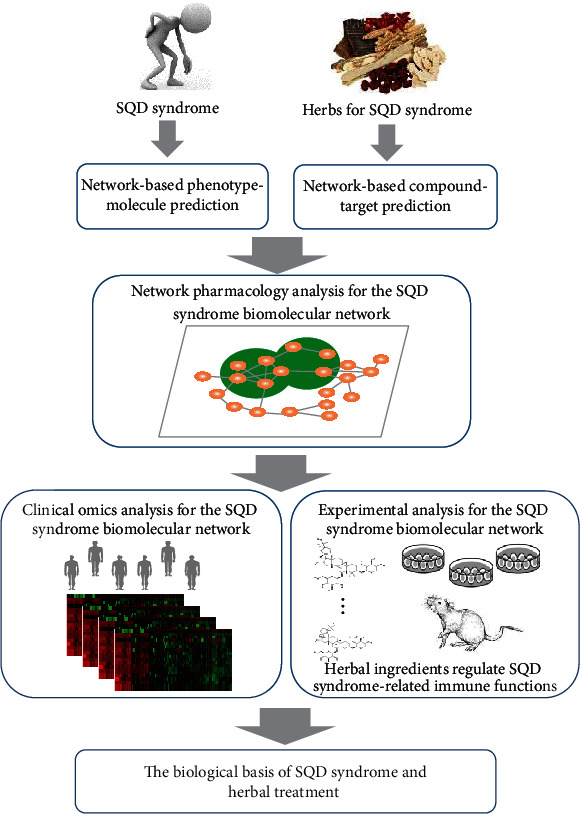
A workflow of the network pharmacology strategy for uncovering the biological basis of SQD syndrome and herbal treatment.

In this article, based on network target theory, we further propose a network pharmacology approach integrating computational, clinical, and experimental investigation to elucidate biological associations between SQD syndromes, diseases, and herbal treatments (Figure 1). First, we computationally predicted the SQD syndrome-related biomolecular network. And then, we further study the biological functions of this network including immune and oxidative stress from clinical transcriptomic data of SQD syndrome-related diseases such as chronic gastritis and hypertension. Furthermore, network pharmacology analysis suggested that the targets of *Radix Astragali*, *Rhizoma Atractylodis Macrocephalae*, *Radix Codonopsis Pilosulae*, *Radix Ginseng*, and *Rhizoma Dioscoreae* commonly used for SQD syndrome are significantly enriched in the SQD syndrome biomolecular network. Experiments further validated that *Radix Astragali* ingredients promoted immune modules of the SQD syndrome biomolecular network. The network pharmacology strategy could provide a new approach to transform experience-based TCM syndrome into biological network-based TCM precision medicine.

## 2. Materials and Methods

### 2.1. Methods for Prediction of the SQD Syndrome Biomolecular Network

In this work, we predicted the SQD syndrome-related biomolecules at the genome-wide level using the CIPHER algorithm with 14 clinical phenotypes of SQD syndrome (Supplementary Table S1). The top 500 biomolecules were selected to form the SQD-related biomolecule list according to the high accuracy of the algorithm. According to the predicted biomolecules, protein-protein interactions, and signaling pathways, we generated the SQD syndrome biomolecular network. CIPHER was a powerful disease gene network-based prediction algorithm [21]. In principle, this algorithm explores the modularity of human phenotype-biomolecule through network-based integration of multiple phenotype similarities among OMIM-recorded diseases and TCM syndromes and protein-protein interactions (PPIs) among candidate biomolecules. This network pharmacology algorithm has been robustly evaluated in the international journal and is used to identify the clinical biomarkers [22, 23].

### 2.2. Computational Validation of the SQD Syndrome Biomolecular Network

In mining for literature on SQD syndrome or compounds, we searched the PubMed database using the keywords “Spleen Qi deficiency syndrome” or the compound name in the abstract and recorded the total number of search results. We downloaded the identified abstracts and extracted the biomolecules listed in the abstracts using a text processing program. For the reliability of CIPHER prediction for SQD syndrome-related biomolecules, we randomly shuffled the protein-protein interaction (PPI) network 1000 times to calculate concordance scores to predict phenotype-gene relationships. The PPI network was generated with 137,037 interactions among 13,388 biomolecules. We selected 500 biomolecules as predicted biomolecules of SQD syndrome in the network each time. The literature mining was performed by the open-source programming language Ruby (version 2.3.0). If a biomolecule cooccurred in the abstract with the SQD syndrome or compound name, we manually verified and considered that the biomolecule is related to SQD syndrome and herbal ingredients. We calculated the recall for predictions and precision as recall = [the intersection of predicted biomolecules and reported biomolecules/reported biomolecules] × 100% and precision = [the intersection of predicted biomolecules and reported biomolecules/predicted biomolecules] × 100%, respectively.

### 2.3. Biological Function Enrichment Analysis of the SQD Syndrome Biomolecular Network

We performed biological function enrichment analysis for predicted biomolecules related to SQD syndrome. The enrichment significance of the predicted biomolecules in the gene sets of GO biological processes or KEGG signaling pathways was determined using Fisher's exact test [24, 25]. Significantly enriched biological processes and pathways (*P* < 0.05, Benjamin correction) were selected for further investigation. The biological function enrichment analysis was performed using open-source programming languages R (version 3.6.1) and Ruby (version 2.3.0).

### 2.4. Clinical Transcriptomic Data Analysis of SQD Syndrome-Related Diseases

We collected gene expression profiles in gastric tissue samples from patients with Cold syndrome (diagnosed as “Spleen Stomach Deficiency Cold (SSDC) syndrome”) or Hot syndrome (diagnosed as “Spleen Stomach Dampness Hot (SSDH) syndrome”) [6]. These samples were assessed using the Affymetrix Human Genome U133 Plus 2.0 Array (U133Plus2.0, Affymetrix, Inc.). The probe signals were generated from the original expression data file via a standardization protocol provided by dChip software [26]. Gene expression data for chronic fatigue syndrome (CFS), irritable bowel syndrome (IBS), and hypertension patients (GSE14577, GSE14841, and GSE75360) were obtained from the GEO database [27]. The selected differentially expressed genes were connected to predicted biomolecules of SQD syndrome by a direct or indirect relationship (including protein-protein interactions or signaling pathway) and related to the enriched biological functions of the SQD syndrome biomolecular network.

### 2.5. Target Prediction of Herbal Ingredients for SQD Syndrome

Firstly, we collected the ingredients of five herbs for SQD syndrome by chemical analysis experiments from literature [28–32], including ingredients from *Radix Astragali*, *Rhizoma Atractylodis Macrocephalae*, *Radix Codonopsis Pilosulae*, *Radix Ginseng*, and *Rhizoma Dioscoreae*. After filtering redundant information, 252 ingredients were obtained. Then, we extracted the chemical structure of each ingredient in the PubChem database for further target prediction by the drugCIPHER algorithm. The ingredients that meet the druglikeness standard (weighted Quantitative Estimate of Druglikeness (wQED) > 0.05) were chosen for further computational analysis. The wQED is calculated by the following formula [33]:

(1) $$\begin{array}{c} \text{wQED}=\exp\left(\frac{\sum_{i=1}^{n}\omega_{i}\ln d_{i}}{\sum_{i=1}^{n}\omega_{i}}\right), \end{array}$$

where *d* is the individual desirability function, *ω* is the weight applied to each function, and *n* is the number of descriptors.

The potential targets of the selected ingredients were predicted by drugCIPHER [34], a network pharmacology algorithm developed for the prediction of compound targets. In principle, by integrating the association of the given compound with FDA-approved drugs and the association of known targets of FDA-approved drugs in the PPI network, drugCIPHER predicts a target list of each compound. The likelihood of the compound-target interaction is defined as

(2) $$\begin{array}{c} \Phi_{p}=\beta_{p}^{\prime }+\underset{dj\in B\left(p\right)}{\sum }\alpha_{pdj}^{\prime }{CS}_{dj}, \end{array}$$

where *d*_*j*_ is the known drug *j* binding to the given protein *p*. *β*′*p* and *α*_*pdj*_′ are the model coefficients. **CS**_*d*_ is the similarity vector of the compound *d* and the known drugs in the algorithm model. According to the concordance score of each compound-target, drugCIPHER prioritizes the proteins in the PPI network, and the candidate proteins with high concordance score *ρ*_*pd*_ are hypothesized to be putative targets of the compound by the following formula:

(3) $$\begin{array}{c} \rho_{pd}=\frac{\mathrm{cov}\left({CS}_{d},\Phi_{p}\right)}{\sigma \left({CS}_{d}\right)\sigma \left(\Phi_{p}\right)}. \end{array}$$

Compounds and proteins with high concordance scores are more likely to exhibit drug-target interactions. The top 100 predicted targets of each compound are selected as potential targets according to the high accuracy of the drugCIPHER algorithm.

Some targets may appear in the target lists of many ingredients in an herb for SQD syndrome. To assess the probability of target proteins being related to the herb for SQD syndrome pharmacological effects, we compared the number of occurrences of each target in the target list of all ingredients. A statistical model was established to compare the number of occurrences of a target protein for each herb with that in a random background; this analysis yielded the significantly frequently occurring targets (*P* < 0.05) as the targets of each herb [35]. The random process is represented by the Poisson binomial statistical model:

(4) $$\begin{array}{c} \mathrm{Pr}\left(K=k\right)=\underset{A\in F_{k}}{\sum }\underset{i\in A}{\prod }p_{i}\underset{j\in A^{c}}{\prod }\left(1-p_{j}\right), \end{array}$$

where Pr(*K* = *k*) is the probability that a protein occurs in the predicted target list of *k* ingredients, *F*_*k*_ is all subsets of *k* ingredients, *A* is one subset of *k* ingredients, and *A*^*c*^ is the complement of subset *A*. In addition, *p*_*i*_ and *p*_*j*_ are the probabilities of a protein being contained in the predicted target list of an ingredient and are calculated as [the number of potential targets/the number of all candidate targets] in the random case. *i* and *j* are used to distinguish different compounds. The *P* value Pr(*K* > *k*) measures the probability of a target occurring in more than *k* ingredients' target lists by 1000 random cases. This adjusted *P* value indicates the significance of targets of herbs for SQD syndrome (*P* value < 0.05 is significant). The target prediction of herbs for SQD syndrome was performed by the opensource programming language R (version 3.6.1).

### 2.6. Experimental Materials and Methods for the SQD Syndrome Biomolecular Network

#### 2.6.1. Reagents

Astragaloside I and astragaloside II were purchased from Nanjing Jingzhu Bio-Technology Co., Ltd. Astragaloside IV (98% purity) was purchased from Chengdu Herbpurify Co., Ltd., and astragalus polysaccharides (70% purity) were purchased from Shanghai Macklin Co., Ltd. The compounds were dissolved in dimethyl sulfoxide (DMSO) (final concentration < 0.1%), which was used as the solvent control for experiments. For experiments, compounds were dissolved in an alkaline solution. Penicillin sodium salt and streptomycin sulfate were purchased from North China Pharmaceutical Co., Ltd. DMEM and trypsin were obtained from Gibco. RPMI-1640 medium was obtained from Beijing Keyin Watson Scientific Development Co., Ltd., and foetal bovine serum was obtained from Beijing Yuanshang Shengma Biotechnology Institute. DMSO was produced by Beijing Chemical Plant. Tetrazolium bromide (MTT) was provided by Beijing Soleble Technology Co., Ltd. ConA and LPS were purchased from Sigma-Aldrich.

#### 2.6.2. Cell Culture

The mouse macrophage cell line RAW264.7 was a gift from Professor Wang Wenjie (Institute of Materia Medica, Chinese Academy of Medical Sciences). The cells were cultured in DMEM supplemented with 10% foetal bovine serum and 100 U/ml penicillin in an incubator containing 5% CO_2_ at 37°C. All cells were digested with 0.25% trypsin-EDTA and passaged twice a week.

#### 2.6.3. Macrophage Proliferation of Herbs for SQD Syndrome

Raw264.7 cells were seeded in a 96-well plate at a concentration of 5 × 10^4^ cells/ml. After a 24 h incubation, the cells were treated with 5 *μ*M or 50 *μ*M astragaloside I (wQED = 0.131), astragaloside II (wQED = 0.131), astragaloside IV (wQED = 0.131), and astragalus polysaccharide (wQED = 0.571). Then, the cells were placed in the incubator for 96 h. Four hours before the end of the incubation, 50 *μ*l of MTT stock solution (2 mg/ml; Soleble Technology, China) was added to each well. After incubation, the cells were pelleted by centrifugation (2000 rpm, 10 min). Then, we gently decanted the cell culture medium and dried the samples with a tissue. Then, 150 *μ*l of DMSO was added to each well. After completely mixing the cells and DMSO by oscillation, the optical density (OD) at 570 nm was measured using an ELISA reader (Bio-Rad, USA).

#### 2.6.4. Spleen Lymphocyte Proliferation of Herbs for SQD Syndrome

Herbs for SQD syndrome, that is, herbs for reinforcing spleen qi such as *Radix Astragali*, were often used to treat colon cancer [36]. Colon cancer has the clinical phenotypes of SQD syndrome. In order to understand the immune regulation of herbs for SQD syndrome, we conducted the spleen lymphocyte proliferation experiment in the SQD syndrome-related colon cancer model. All animal protocols conformed to the Guidelines for the Care and Use of Laboratory Animals and were approved by the Animal Care and Use Committee of the Chinese Academy of Medical Sciences and Peking Union Medical College, and Balb/c mice were purchased from Beijing Weitong Lihua Experimental Animal Technology Co., Ltd. (No. SCXK2012-0001). Balb/c female mice were housed in a controlled environment at 18~25°C with 50% to 70% relative humidity. C26 cells were preserved by the Department of Pharmacology, Institute of Materia Medica, Chinese Academy of Medical Sciences and Peking Union Medical Colleges. Actively growing tumour tissues were dissected, cut, and ground to generate a tumour cell suspension in sterile physiological saline (5 × 10^7^ cells/ml). Each mouse was inoculated on the back with 0.2 ml of the cell suspension. The day after inoculation, Balb/c mice were randomly divided into two groups and administered drugs once a day for 12 days. To illustrate the herbal treatment for regulating the lymphocyte function module of the SQD syndrome biomolecular network, one group received saline as a negative control, and the other group was treated with 50 mg/kg astragaloside IV according to the results of preliminary experiments on mice.

After treatment with astragaloside IV, the mice were sacrificed by cervical dislocation, and the spleens were removed under aseptic conditions and ground in a mortar using a 1 ml sterile syringe. The cell suspension was then transferred to a plastic centrifuge tube and pelleted. The cells were diluted to 1 × 10^7^ cells/ml, and 100 *μ*l/well cell suspension was seeded in a 96-well cell culture plate (with 3.0 *μ*g/ml ConA or 5.0 *μ*g/ml LPS). The cell culture plate was placed in the incubator at 37°C with 5% CO_2_ for 48 h. Four hours before the end of the incubation, 50 *μ*l of MTT solution (2 mg/ml; Soleble Technology, China) was added to each well. Then, the plates were centrifuged at 2000 rpm for 10 min to pellet the cells, and the remaining MTT solution was removed with a tissue. A total of 150 *μ*l of DMSO was added to each well. Following complete mixing, the OD at 570 nm was measured using an ELISA reader (Bio-Rad, USA).

### 2.7. Statistical Analysis of the SQD Syndrome Biomolecular Network

The statistical significance was performed using Student's *t*-test via GraphPad Prism 5.0 in clinical transcriptional profiles and experiments. Data are expressed as the mean ± standard deviation in experiments. Enrichment analysis was conducted by Fisher's exact test via programming languages R (version 3.6.1) and Ruby (version 2.3.0). *P* values less than or equal to 0.05 were considered significant.

## 3. Results

### 3.1. Computational Analysis of the SQD Syndrome Biomolecular Network

**Figure 2:**
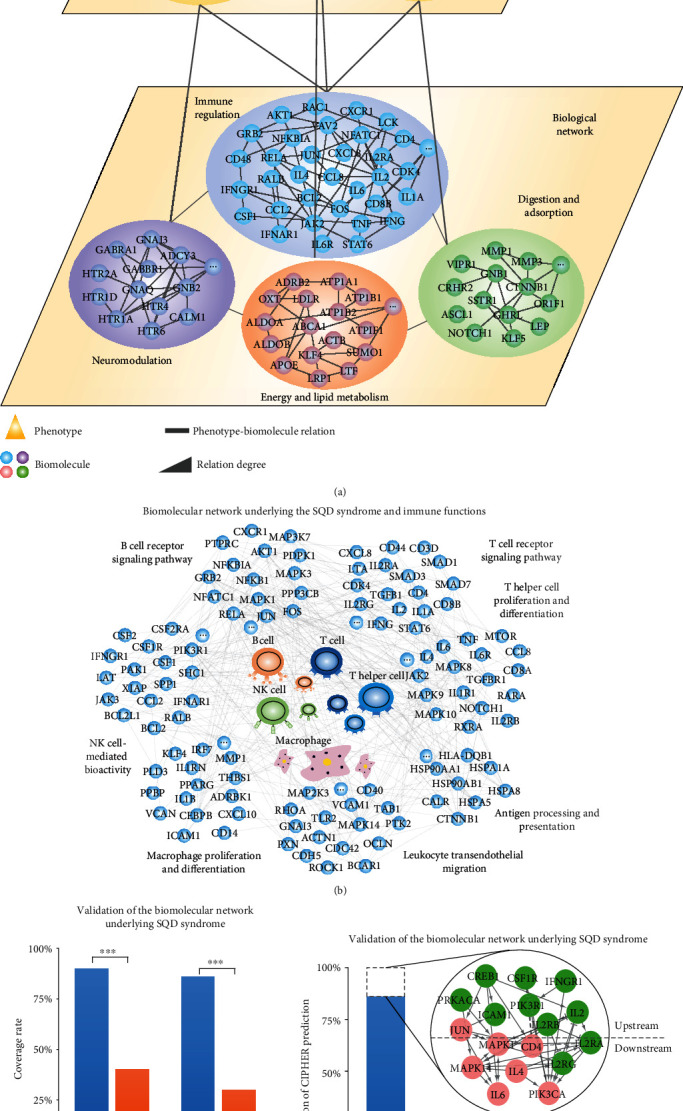
The biomolecular network underlying SQD syndrome was constructed based on clinical phenotypes and protein-protein interactions. (a) SQD syndrome phenotype-biomolecule comodule network. (b) SQD syndrome immune biomolecular network (biomolecules with significant enrichment in immune biological processes and pathways are marked, *P* < 0.05). (c) Validation of the SQD syndrome biomolecular network. (d) The predicted biomolecules of SQD syndrome that are not reported in the literature are upstream or in the same pathway.

We predicted the SQD syndrome biomolecular network at the genome-wide level using the CIPHER method with 14 clinical phenotypes of SQD syndrome (Supplementary Table S1). The top 500 biomolecules were predicted to form the SQD-related biomolecular network according to the high accuracy of the CIPHER algorithm [21]. As listed in Table 1, these biomolecules are significantly enriched in biological pathways and biological processes including immunity, metabolism, endocrine biological functions such as the T cell receptor signaling pathway, response to oxidative stress, and regulation of the lipid metabolic process. The results indicated that the biological basis of SQD syndrome is closely related to the above biological processes, as shown in Figure 2(a).

The enrichment results of the SQD syndrome biomolecular network were further validated by literature mining. We searched for the relationship between enriched biological functions and SQD syndrome in the literature. Patients with SQD syndrome may exhibit immune disorders. We used the immune modules of the SQD syndrome-related network as a starting point to systematically understand the immune-related biological basis of SQD syndrome (Figure 2(b)). The precision and recall rate of the predicted biomolecules related to SQD syndrome predicted by CIPHER were calculated for the validation via literature mining. The precision and recall rates of the predicted biomolecules related to SQD syndrome were significantly higher than those of the randomly selected biomolecules (Figure 2(c)). The top 20 nodes with the highest degrees in the immune biomolecular network of SQD syndrome and their biological functions are listed in Table 2. As shown in Figure 2(d), the predicted molecules related to SQD syndrome that are not referenced in the literature are upstream or in the same pathway. The SQD syndrome biomolecular network provides scientific support for further unveiling the biological basis of SQD syndrome.

### 3.2. Decreased Innate Immune Modules of the SQD Syndrome Biomolecular Network in Transcriptional Profiles of Chronic Gastritis Patients and IBS Patients

**Figure 3:**
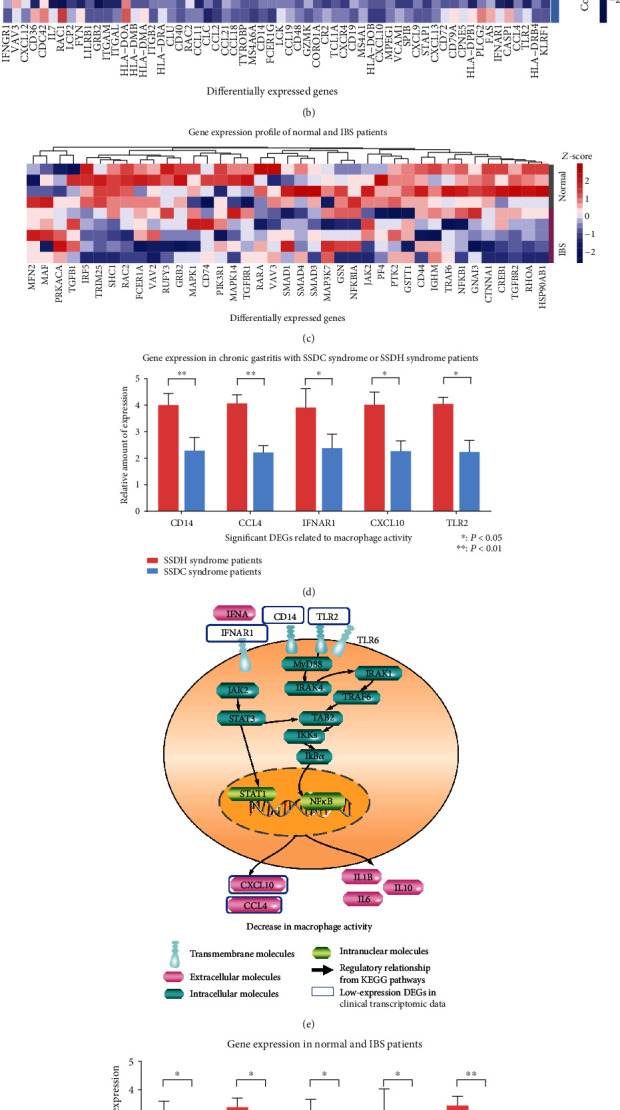
The immune molecular network of SQD syndrome covers the DEGs in immune function in chronic gastritis patients and IBS patients. (a) DEGs in chronic gastritis patients with SSDC syndrome and DEGs in IBS patients in the SQD syndrome biomolecular network. (b) The expression of the selected genes in patients with chronic gastritis with SSDC syndrome or SSDH syndrome. (c) The expression of the selected genes in normal individuals and IBS patients. (d) Gene expression related to macrophages in chronic gastritis with SSDC syndrome. (e) DEGs in chronic gastritis with SSDC syndrome in the immune molecular network of SQD syndrome that reduce macrophage function. ^∗^*P* < 0.05, ^∗∗^*P* < 0.01: compared with chronic gastritis patients with SSDH syndrome. (f) Gene expression related to NK cell-mediated bioactivity in IBS patients. (g) DEGs in IBS patients that regulate the NK cell pathway in the SQD syndrome biomolecular network of SQD syndrome. ^∗^*P* < 0.05, ^∗∗^*P* < 0.01: compared with the normal group.

According to the holistic perspective of TCM, SQD syndrome is involved in both local and holistic disorders, which is closely related to many diseases. Literature mining was conducted to identify SQD syndrome-related diseases in the abstracts of the China National Knowledge Infrastructure (CNKI) database. Chronic gastritis and irritable bowel syndrome (IBS) are digestive diseases associated with SQD syndrome. We analyzed gene expression profiles in tissue samples from chronic atrophic gastritis (CAG) patients with the Cold syndrome (diagnosed as SSDC syndrome) or the Hot syndrome (diagnosed as SSDH syndrome) and IBS patients or normal individuals. SSDC syndrome and IBS are closely related to SQD syndrome which exhibits similar phenotypes (e.g., boredom, fatigue, lassitude, and loose stools). DEGs for chronic gastritis with the Cold syndrome and IBS patients in the SQD syndrome biomolecular network were involved in immune functions and response to oxidative stress (Figure 3(a)). DEG analysis revealed disorders in several immune functions and oxidative stress in chronic gastritis and irritable bowel syndrome (Figures 3(b) and 3(c)). In terms of immune functions, macrophage proliferation-related genes of the SQD syndrome biomolecular network were expressed at low levels in chronic gastritis patients with Cold syndrome by integrating the transcriptome data (Figures 3(d) and 3(e)). In the transcriptomic data of IBS patients, gene expression involved in NK cell activity was decreased in the network (Figures 3(f) and 3(g)). In terms of oxidative stress, the network node JAK2 mediates oxidative stress associated with the JAK-STAT signaling pathway and GSTT1 regulates the glutathione metabolic process to perform oxidative stress activity. By integrating the network analysis and transcriptional profile, we illustrated dysfunctions of the innate immune and oxidative stress modules in the SQD syndrome biomolecular network.

### 3.3. Reduced Lymphocyte Activities of the SQD Syndrome Biomolecular Network in Transcriptional Profiles of Patients with CFS and Patients with Hypertension

**Figure 4:**
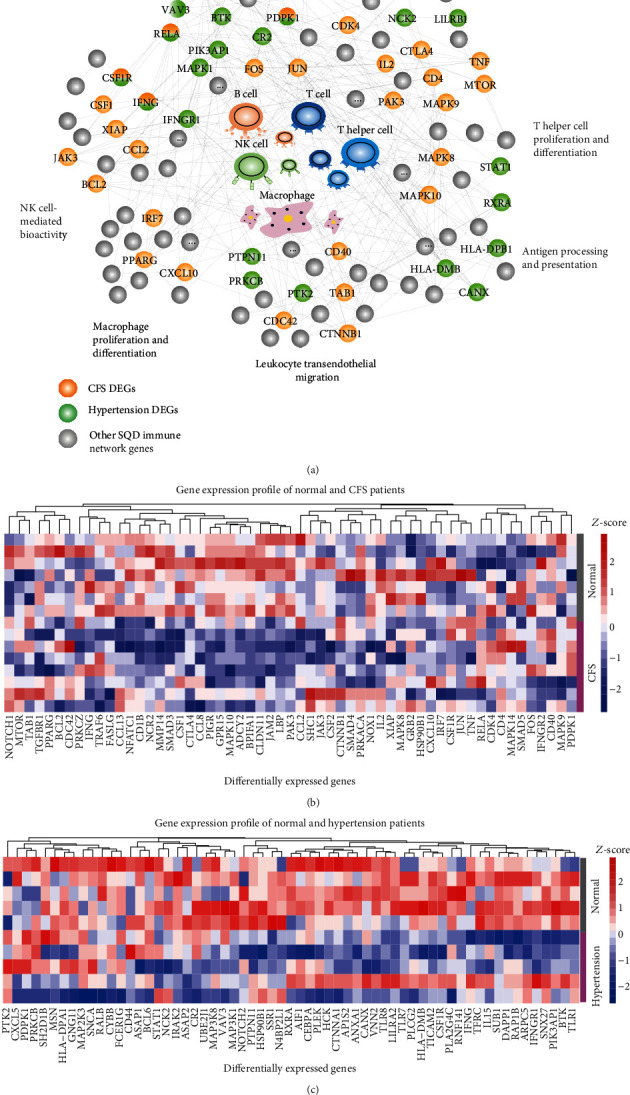
Immune biomolecular network of SQD syndrome contains immune function of SQD syndrome-related diseases. (a) DEGs in CFS or hypertension in the immune molecular network of SQD syndrome. (b) The expression of the selected genes in normal individuals and patients with CFS. (c) The expression of the selected genes in normal individuals and patients with hypertension.

**Figure 5:**
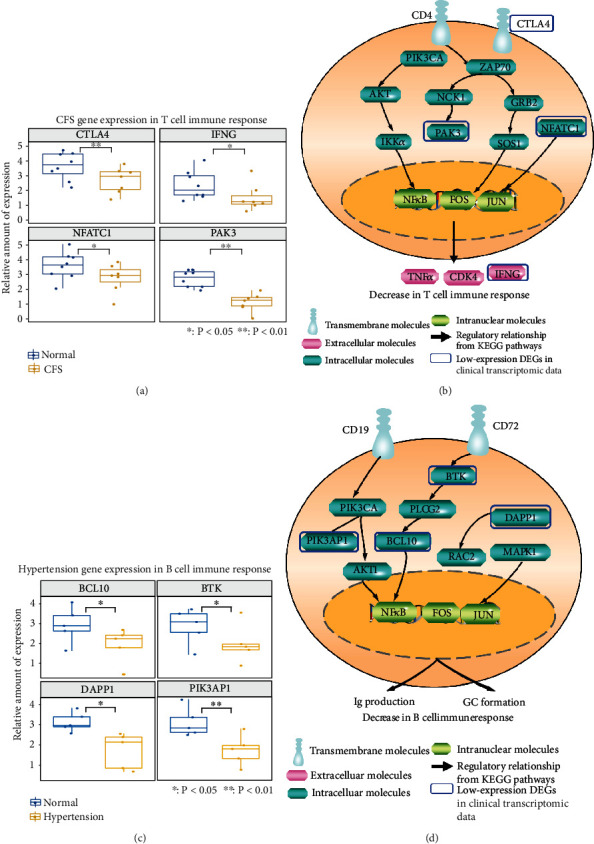
(a) Selected gene expression in normal individuals and CFS patients. (b) DEGs in CFS in the immune molecular network of SQD syndrome that reduced T cell immune function. (c) Selected gene expression in normal individuals and hypertension patients. (d) DEGs in hypertension in the immune molecular network of SQD syndrome that reduced B cell immune function. ^∗^*P* < 0.05, ^∗∗^*P* < 0.01: compared with the normal group.

SQD syndrome is also a kind of the whole body's dysfunctions. According to the holistic perspective of TCM, SQD syndrome not only refers to local digestive disease but also contains the whole body's dysfunctions such as chronic fatigue syndrome (CFS) and hypertension. CFS and hypertension have some clinical manifestations of SQD syndrome [7, 37]. Therefore, in order to investigate the holistic biological basis of SQD syndrome, we analyzed gene expression profiles associated with CFS and hypertension for illustrating biological features of the SQD syndrome biomolecular network. The immune-related DEGs in these diseases were significantly covered by the SQD syndrome immune biomolecular network (*P* < 0.05). Immune-related DEGs in CFS and hypertension were present in the SQD syndrome immune biomolecular network (Figure 4(a)). Analysis of differential gene expression revealed several immune dysfunctions in chronic fatigue syndrome (Figure 4(b)) and hypertension (Figure 4(c)). Some genes with decreased expression in CFS patients are significantly related to T cell function (Figure 5(a)). For example, low expression of NFATC1 regulates IFNG and other genes to reduce the adaptive immune response (Figure 5(b)). In addition, downregulated genes in hypertension patients are related to B cell function, as shown in Figure 5(c). Reductions in VAV3 and other genes in the B cell receptor signaling pathway lower Ig production (Figure 5(d)). The above results suggested a reduction in adaptive immune functions in SQD syndrome.

### 3.4. Network Pharmacology Analysis of Herbal Treatment for SQD Syndrome

**Figure 6:**
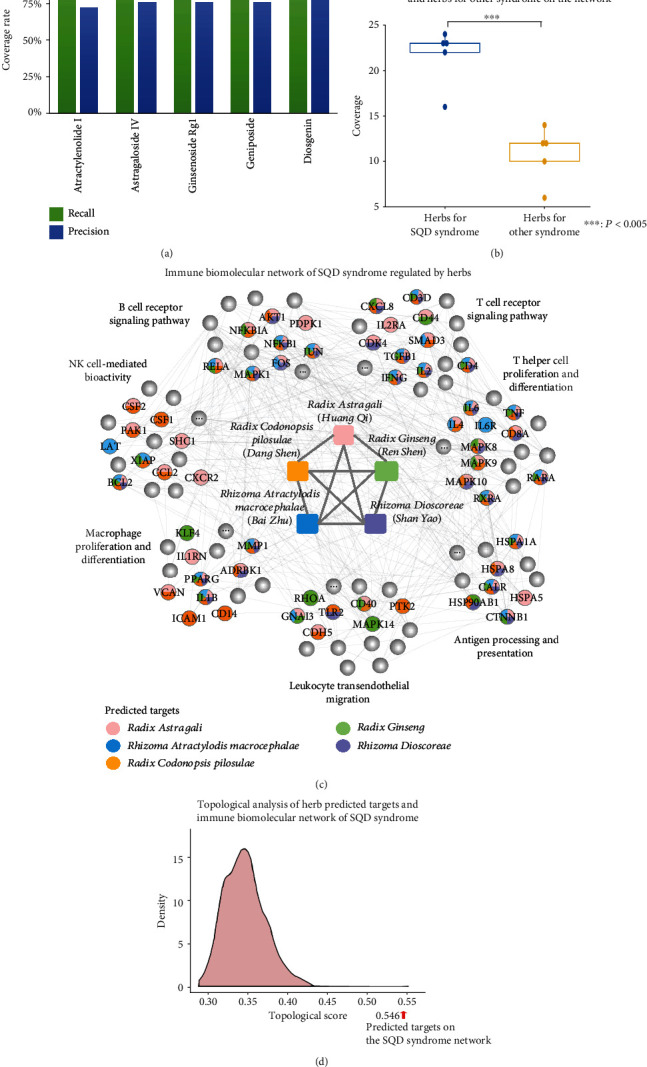
The immune biomolecular network of SQD syndrome uncovers the mechanisms of action of commonly used herbs for SQD syndrome. (a) Validation of the drugCIPHER-predicted targets of compounds in herbs for SQD syndrome. (b) The predicted targets of herbs for SQD syndrome were significantly enriched in the immune biomolecular network of SQD syndrome compared to herbs for other syndromes. (c) The SQD syndrome immune molecular network covers the predicted targets and the features of herbs for SQD syndrome. (d) The predicted targets of herbs for SQD syndrome are closely related to the immune biomolecular network of SQD syndrome in the topological structure compared to the random situation.

SQD syndrome exhibits its specific phenotypes and is closely linked to herbs for reinforcing spleen qi. Therefore, SQD syndrome is not only related to phenotypes but also associated with herbal treatment. Five herbs were commonly used for phenotypes associated with SQD syndrome in the CNKI database, that is, herbs for invigorating spleen and tonifying spleen qi such as *Rhizoma Atractylodis Macrocephalae*, *Radix Astragali*, *Radix Codonopsis Pilosulae*, *Radix Ginseng*, and *Rhizoma Dioscoreae*. These herbs are also used to treat SQD syndrome-related diseases [11, 38–40]. Herbal ingredients tend to bind multiple targets. Thus, we sought to identify the targets regulated by the herbal ingredients and to understand the relationships between the predicted targets [18, 41]. We validated the reliability of the predicted targets by literature mining on herbal ingredients for SQD syndrome with greater than 100 literature records. As shown in Figure 6(a), greater than 70% of the biomolecules related to representative herbal ingredients with literature evidence were linked to potential targets via direct mapping or indirect connections, such as PPIs or signaling pathways. The results show that the predicted targets can help to describe the mechanisms of these herbs for SQD syndrome.

The SQD syndrome immune biomolecular network can be applied to distinguish herbs for SQD syndrome and those for other syndromes. Here, five herbs with no literature evidence for treating SQD syndrome in the CNKI database (*Radix Isatidis*, *Cassia Seed*, *Swertia*, *Herba Lysimachia*, and *Folium Isatidis*) were selected as herbs for syndromes other than SQD syndrome (Figure 6(b)). The biomolecules related to herbs for SQD syndrome covered the network (Figure 6(c)). Moreover, the topological relationship between the predicted targets of herbs for SQD syndrome and the network was measured by NIMS, a network-based topological analysis method [42]. The analysis results revealed a significant correlation between the predicted targets of herbs for SQD syndrome and the immune biomolecular network (Figure 6(d)). As shown in Supplementary Table S2, the target lists of herbal ingredients for SQD syndrome are covered in the SQD syndrome immune biomolecular network. This network uncovers the immune regulation effects of herbs for SQD syndrome.

### 3.5. Experimental Validation of Herbal Ingredients for Immune Modules of the SQD Syndrome Biomolecular Network

**Figure 7:**
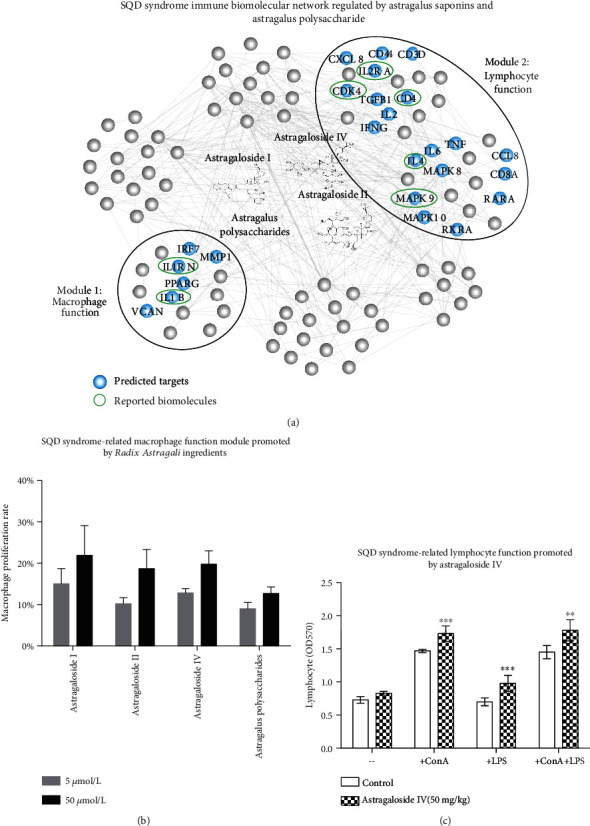
Ingredients in herbs for SQD syndrome may exert immune-enhancing activity related to the immune biomolecular network of SQD syndrome. (a) The predicted targets of the representative ingredients in *Radix Astragali* may regulate immune function in the macrophage and lymphocyte function modules in the SQD syndrome biomolecular network. (b) Representative ingredients in *Radix Astragali* improve macrophage proliferation. (c) Astragaloside IV promotes the proliferation of spleen lymphocytes. ^∗∗^*P* < 0.01, ^∗∗∗^*P* < 0.005: compared with the control group.

According to the traditional efficacy of Chinese medicine in the Compendium of Materia Medica, *Rhizoma Atractylodis Macrocephalae* is used for invigorating the spleen to eliminate dampness and *Radix Astragali* is the most common herb for tonifying spleen qi. Based on the traditional efficacy of Chinese medicine and literature mining for treating SQD syndrome, *Radix Astragali* is suitable to regulate the SQD syndrome-related immune functions [43]. Astragalus saponin ingredients and astragalus polysaccharides are the representative ingredients of an herb for SQD syndrome, *Radix Astragali*. The predicted targets of these ingredients were related to the SQD syndrome biomolecular network (Figure 7(a)). In order to confirm that the immune regulation of the representative ingredients of herbs for SQD syndrome is revealed in the SQD syndrome biomolecular network, we find experimental evidence for immune regulation of some ingredients in literature and further conducted pharmacological experiments. Experimental results illustrated that astragaloside saponin ingredients and astragalus polysaccharide have immunoregulation of pharmacological activities *in vivo* and *in vitro*. For example, astragaloside saponin ingredients and astragalus polysaccharides have strong promoting effects on the phagocytosis of macrophages [44]. Astragaloside IV could increase T and B lymphocyte proliferation and antibody production [45]. Astragalus saponin ingredients (astragaloside I, astragaloside II, and astragaloside IV) and astragalus polysaccharides promote the proliferation of macrophages (Figure 7(b)). Experimental results demonstrated that astragaloside IV alone could significantly enhance the proliferation of splenic lymphocytes with or without ConA or LPS stimulation and the effects were stronger than those of the vehicle control (Figure 7(c)). The experimental results further demonstrated that astragalus saponin ingredients and astragalus polysaccharides enhance immune regulation by macrophage and lymphocyte modules in the SQD syndrome biomolecular network.

## 4. Discussion

Traditional Chinese medicine states that “pathogenic-qi cannot invade the body if “health-qi” remains strong” in *Plain Questions about Huangdi Neijing*. “Spleen qi” is an important category of “health qi.” The biological basis of SQD syndrome is unclear, and the associated mechanisms of disease-syndrome-herb are not known. This situation has caused certain difficulties for clinical diagnosis and herbal treatment. Rats treated with reserpine, which exhibit similar signs to SQD syndrome, exhibit neuro-endocrine-immune disorders [46]. In hypertension, a deficiency in macrophage function and B cell dysfunction induced blood pressure elevation and vascular injury [47]. As shown in our results, SQD syndrome-related biological functions are also associated with oxidative stress. The antioxidant module of the SQD syndrome biomolecular network decreases in transcriptional profiles of patients with chronic gastritis, which leads to oxidative stress. Oxidative stress could reduce the activity of NK cells and T lymphocytes [48]. Increased oxidative stress damage and insufficient immune regulation lead to cellular dysfunction causing aging [49].

Given that disease is a multifactorial consequence, the corresponding drug combinational therapy regulates multiple targets to produce therapeutic effects. SQD syndrome is related to the reduction in immune functions and antioxidant activity [50]. The molecular mechanisms of herbal formula in treating diseases and syndromes are closely related. For example, we used a heterogeneous biological network of the association of disease-SQD syndrome-herb to reveal relevant molecular mechanisms. *Radix Astragali* treats SQD syndrome-related diseases by improving immune function and inhibiting oxidative stress [51, 52]. The experimental results also demonstrated that astragaloside I, astragaloside II, astragaloside IV, and astragalus polysaccharides may increase the proliferation ability of the macrophage and lymphocyte in the SQD syndrome bimolecular network. The ingredients in *Radix Ginseng* regulate a portion of the biological functions including NK cell activity and antioxidant activity in the SQD syndrome bimolecular network [53, 54].

Research on SQD syndrome by systems biology approaches, including metagenomics, has been increasingly emerging [55]. Network pharmacology studies have investigated the biological basis of TCM syndrome from the perspective of biomolecular networks [18]. This is a promising approach that is consistent with the features of TCM syndrome. The efficacy of the SQD syndrome immune network can be attributed to the following aspects. First, the integration of phenotypes and biomolecular networks helps identify the biological basis of SQD syndrome, which could contribute to the development of effective treatments. Second, the biomolecular network contains the biological features of differentially expressed genes in SQD syndrome-related diseases and herbal treatment. However, there are some limitations of the present study. The identified immune functions in the SQD syndrome-related network need to be further verified in large clinical samples. Further investigation should be conducted to detect different tissues in order to analyze the tissue specificity of SQD syndrome. Besides, the herbal ingredients for SQD syndrome used in the present study are still incomplete. Other herbs for SQD syndrome and more ingredients *in vivo* and *in vitro* need to be further collected.

## 5. Conclusion

In summary, this study proposed a novel network pharmacology strategy to predict the SQD syndrome biomolecular network. The clinical transcriptomic data and pharmacological experiments further evaluated and validated the biological features of the SQD syndrome-related network. TCM is a type of personalized medicine with long-term clinical practice experience. This study used SQD syndrome as an example to uncover the biological basis of SQD syndrome from the network pharmacology perspective, thereby providing a way for the precision diagnosis and treatment of SQD syndrome-related diseases such as chronic gastritis and hypertension. Based on the network pharmacology approach, this study not only reveals parts of the biological basis of SQD syndrome but also provides a novel insight for exploring the mechanisms of SQD syndrome-related diseases.

## Figures and Tables

**Table 1:** Several enriched biological processes and pathways of the SQD syndrome biomolecular network.

Class	Biological process and pathway	*P* value
Immune system	T cell receptor signaling pathway	4.2*E* − 15
	Helper T cell proliferation and differentiation	3.2*E* − 15
	Macrophage proliferation and differentiation	5.6*E* − 11
	Antigen processing and presentation	3.2*E* − 4
	Leukocyte transendothelial migration	1.3*E* − 4
	B cell receptor signaling pathway	6.3*E* − 4
	Natural killer cell-mediated bioactivity	0.0054

Endocrine and metabolism system	Estrogen signaling pathway	1.2*E* − 16
	Response to oxidative stress	7.2*E* − 7
	Regulation of the lipid metabolic process	5.3*E* − 14

Nervous system	Neurotrophin signaling pathway	7.4*E* − 12
	Dopaminergic synapse	8.7*E* − 5

Digestive system	Digestion	0.0058
	Regulation of the digestive system process	0.0084

**Table 2:** Immune-related nodes in the SQD syndrome biomolecular network.

Top 20 biomolecules	Degree	Biological process and pathway
PIK3R1	29	T cell receptor signaling pathway
MAPK14	27	T cell receptor signaling pathway
LCK	27	Natural killer cell-mediated bioactivity
JUN	26	B cell receptor signaling pathway
GRB2	25	T cell receptor signaling pathway
MAPK1	24	T cell receptor signaling pathway
AKT1	24	B cell receptor signaling pathway
MAPK8	24	Helper T cell proliferation and differentiation
SMAD3	23	T cell receptor signaling pathway
FYN	23	Natural killer cell-mediated bioactivity
RELA	23	B cell receptor signaling pathway
HSP90AA1	23	Antigen processing and presentation
CTNNB1	21	Antigen processing and presentation
SHC1	20	Natural killer cell-mediated bioactivity
TGFBR1	20	Helper T cell proliferation and differentiation
JAK2	17	Helper T cell proliferation and differentiation
FOS	17	B cell receptor signaling pathway
MAP3K7	17	B cell receptor signaling pathway
NFKB1	17	Leukocyte transendothelial migration
MAPK3	16	T cell receptor signaling pathway

## Data Availability

Further information and requests for data may be directed to and will be fulfilled by the lead contact Shao Li (shaoli@tsinghua.edu.cn) on a reasonable request.

## Abbreviations

TCM: Traditional Chinese medicine
SQD: Spleen qi deficiency
SSDC: Spleen stomach deficiency Cold
SSDH: Spleen stomach dampness Hot
IBS: Irritable bowel syndrome
CFS: Chronic fatigue syndrome
DEGs: Differentially expressed genes
wQED: Weighted quantitative estimate of druglikeness
DMSO: Dimethyl sulfoxide.

## Supplementary Materials

Supplementary Materials Table S1: clinical phenotypes of spleen qi deficiency syndrome. Table S2: SQD syndrome biomolecular network regulated by partial ingredients in herbs for reinforcing spleen qi.
