# Supplementary material for: Network Pharmacology to Uncover the Biological Basis of Spleen Qi Deficiency Syndrome and Herbal Treatment
Source: Oxid Med Cell Longev. 2020 Aug 27;2020:2974268. doi: 10.1155/2020/2974268 (PMC7474375; doi:10.1155/2020/2974268)
Supplement: Supplementary Materials — Table S1: clinical phenotypes of spleen qi deficiency syndrome. Table S2: SQD syndrome biomolecular network regulated by partial ingredients in herbs for reinforcing spleen qi. [file 2974268.f1.docx]

**Table S1: Clinical phenotypes of spleen-qi-deficiency syndrome**

| **Clinical phenotype** | **Reference** |
| --- | --- |
| Nervous fatigue | [1] |
| Boredom | [1] |
| Hypodynamia | [1] |
| Laziness to speak | [1] |
| Loose stool | [1] |
| Abdominal distension | [1] |
| Poor appetite | [1] |
| Sallow complexion | [1] |
| Emaciation | [2] |
| Upset stomach | [1] |
| Nausea and vomiting | [1] |
| Inability to defecate | [2] |
| Sleeplessness | [2] |
| Bowel sounds | [2] |

**References:**

[1] Zhang S, Hu L, Li R. Expert consensus on tcm diagnosis and treatment of spleen deficiency syndrome. Journal of Traditional Chinese Medicine. 2017; 58: 1525-30.

[2] Lin J, Hu J, Liu B. Research on conceptual framework of pro scale in spleen-qi deficiency syndrome. Tianjin Journal of Traditional Chinese Medicine. 2013; 30: 277-81.

**Table S2: Immune biomolecular network of spleen-qi-deficiency syndrome regulated by partial ingredients in herbs**

| **Compound** | **DrugCIPHER partial target** | **Reported pharmacological activity** | **Reference** |
| --- | --- | --- | --- |
| Astragalus polysaccharide | IL6, IL1B | Promotion of macrophage proliferation | [1] |
| Astragalus polysaccharide | IFNG, CDK4 | Regulation of T cell subsets | [2] |
| Astragaloside | IL6, TNF | Promotion of T cell proliferation and differentiation | [3] |
| Ginsenoside rh2 | IL2, CCL2 | NK cell activity increased | [4] |
| Ginseng polysaccharides | PPARG, IL1B | Macrophage phagocytosis increased | [5] |
| Atractylodes macrocephalaon polysaccharide | HSPA1A, RXRA | Peripheral blood leukocyte level increased | [6] |
| Chinese yam polysaccharide | CD8A, MAPK8 | Promotion of T cell proliferation | [7] |
| Codonopsis pilosula polysaccharide | MAPK9, IL6 | Macrophage phagocytosis increased | [8] |

**References:**

[1] Xu HD et al. Effects of Astragalus polysaccharides and astragalosides on the phagocytosis of Mycobacterium tuberculosis by macrophages. J Int Med Res 2007; 35: 84-90.

[2] Hou YC et al. Modulatory Effects of Astragalus Polysaccharides on T-Cell Polarization in Mice with Polymicrobial Sepsis. Mediat Inflamm, 2015: 826319.

[3]Li Y et al. Immune regulation mechanism of Astragaloside IV on RAW264.7 cells through activating the NF-κB/MAPK signaling pathway. International Immunopharmacology 2017; 49: 38-49.

[4]Wang M et al. Ginsenoside Rh2 enhances the antitumor immunological response of a melanoma mice model. Oncol Lett 2017; 13: 681-685.

[5]Lim TS et al. Immunomodulating activities of polysaccharides isolated from Panax ginseng. J Med Food 2004; 7: 1-6.

[6]Hu XL et al. Ettects of Atractylodes macrocephala koidz and PAM on the growth performance and immune function of SD rats. Chin J Vet Med, 2006, 40(1):2-6.

[7]Zhao GH et al. Effects of chinese yam polysaccharide(rdps-i) on immune function of cancer bearing mice. Acta Nutrimenta Sinica, 2003, 25(1): 110-112.

[8]Qin, T. et al. Effects of Selenizing Codonopsis pilosula Polysaccharide on Macrophage Modulatory Activities. J Microbiol Biotechn 2016; 26: 1358-1366.
